# Root resorption of primary molars and dental development of premolars in children with Osteogenesis Imperfecta medicated with bisphosphonates, grouped according to age and gender

**DOI:** 10.1186/s12903-024-04557-3

**Published:** 2024-07-28

**Authors:** Clara Sandibel Garcete Delvalle, M. Joaquín De Nova García, María Rosa Mourelle Martínez

**Affiliations:** Faculty of Dentistry, Complutense University of Madrid, Madrid, Spain

**Keywords:** Osteogenesis imperfecta, Bisphosphonates, Root resorption, Dental development, Premolar, Primary molar, Panoramic radiograph

## Abstract

**Background:**

Osteogenesis imperfecta (OI) is an inherited disorder characterized by bone fragility and skeletal alterations. The administration of bisphosphonates (BPs) to patients with OI reduces pain, thereby improving their quality of life. The main mechanism of action of BPs is the inhibition of osteoclast action. In the oral cavity of children with OI during growth and development, physiological processes that require the function of osteoclasts occur. The aim of this investigation was to study the dental development of premolars and the root resorption of primary molars in children with OI medicated with BPs according to age and sex.

**Methods:**

An observational and analytical study was designed. The study sample consisted of 26 6- to 12-year-old children with a confirmed diagnosis of OI treated with BPs with available panoramic radiographs. The control group consisted of 395 children with available panoramic radiographs. Both groups were divided into subgroups according to sex and age. The third quadrant was studied, focusing on the first left temporary molar (7.4), the second left temporary molar (7.5), the first left permanent premolar (3.4) and the second left permanent premolar (3.5). The *Demirjian* method was used to study the *dental development* of 3.4 and 3.5, and the *Haavikko* method was used to study the *root resorption* of 7.4 and 7.5. The Mann‒Whitney U test was used for comparisons, and *p* < 0.05 indicated statistical significance.

**Results:**

The mean chronological age of the 421 patients was 9.21 years (95% CI 9.05–9.37). The sample was reasonably balanced by sex, with 52.5% (221 patients) boys versus 47.5% (200 patients) girls. Delayed exfoliation and tooth development were described in children with OI (*p* = 0.05). According to sex, the root resorption of primary molars and tooth development were significantly lower in boys in both groups and in girls in the OI group, but the differences between the age groups were not significant.

**Conclusions:**

Children with OI treated with BPs exhibit delayed dental development of the premolars and delayed root resorption of the primary molars. Boys exhibited delays in both variables, but the differences by age subgroup were not significant. These clinical findings support the importance of clinically and radiographically monitoring the dental development and root resorption of primary teeth in children with OI treated with BPs to avoid alterations of the eruptive process.

**Supplementary Information:**

The online version contains supplementary material available at 10.1186/s12903-024-04557-3.

## Background

Osteogenesis imperfecta (OI) is an inherited disorder characterized by bone fragility and skeletal alterations [1]. In the vast majority of cases, OI is caused by a mutation in one of the two genes that encode type I collagen (COL1A1 or COL1A2). In recent years, mutations in other genes that could cause OI have also been identified [2, 3].

Typical manifestations of OI include blue sclera, hearing loss, joint hypermobility, growth delay, and bone pain. Dental alterations including dentinogenesis imperfecta, agenesis, oligodontia and malocclusions have been described [4–9].

The main treatment for OI is increasing bone density [10]. The administration of bisphosphonates (BPs) to patients with OI reduces pain, thereby improving their quality of life [11, 12]. The main mechanism of action of BPs is to inhibit osteoclast action and bone resorption [12, 13]. 

The development of dental eruption involves complex processes, such as resorption of the primary dentition, a physiological process that is part of the growth and development of the child. This process allows for exfoliation of the primary dentition and eruption of the permanent dentition. The roots of primary teeth are resorbed when active eruption of the permanent tooth begins, and the dental follicle approaches the root surface. The chronology and coordination of these processes allows the growth and development of the stomatognathic system [14–16].

Studies in experimental animals have indicated that BPs alter tooth eruption and root formation [17, 18] and induce dental malformations [19]. A few studies in children with OI have shown that tooth development, resorption of the primary tooth and eruption of the permanent tooth are altered. Notably, the studies by Vuorimeis et al. [20] and Malmgren et al. [21] are the only two studies that have previously analysed the eruptive process and compared OI patients medicated with BPs with unmedicated OI patients and a healthy control group [20].

In the oral cavity of children with OI, during growth and development, physiological processes that require the function of osteoclasts occur.

Physiological processes such as tooth eruption are carried out at the expense of clastic cells. The administration of BPs during growth and development could alter the exfoliation of the primary dentition and dental development, thus affecting the entire eruptive process. The aim of this investigation was to study the dental development of premolars and the root resorption of primary molars in children with osteogenesis imperfecta medicated with BPs according to age and sex.

## Methods

The research was carried out at the Complutense University of Madrid (UCM) in the Faculty of Dentistry with the support of the AHUCE Foundation under the collaboration agreement between the AHUCE Foundation and UCM. A cross-sectional, observational and analytical study was designed.

For dental diagnosis, clinical history data and panoramic radiographs are routinely requested.

### Sample characteristics

The study group consisted of patients with osteogenesis imperfecta medicated with BPs, while the control group included healthy patients without systemic diseases. The study group consisted of 26 6- to 12-year-old children with a confirmed diagnosis of osteogenesis imperfecta treated with BPs with available panoramic radiographs. As OI is a rare disease with an incidence ranging between 6 and 20 cases per 100,000 live births, a calculation of the needed sample size could not be carried out [1]. A study conducted using a human sample was used as a reference. This study investigated dental development, root resorption and the emergence of permanent teeth in patients with OI treated with BPs and included a sample of 25 patients with OI [20].

The participants were classified into groups based on sex and age. The two age groups included patients aged 6-8.9 years and patients aged 9–12 years.

Special attention was given to ensure that the control group sample was as similar as possible to the study group in regard to age and sex for comparability. The control group sample consisted of 395 children with available panoramic radiographs and were divided into subgroups according to sex and age. The two age groups included younger patients aged 6–9 years and older patients aged 9–12 years. At no time was there any direct contact with the children since the study was carried out by analysing panoramic X-rays and medical history data.

Chronological age was calculated up to two decimal points for each subject by subtracting the date of birth from the date the radiograph was taken after age conversion to decimals.

**The inclusion criteria of the study group are described below**:

- Diagnosis of osteogenesis imperfecta (OI).
- Age between 6 and 12 years.
- Complete and up-to-date medical history, including the type of OI, the type of antiresorptive agent, weight and the administration period.
- Period of administration of the antiresorptive agent equal to or greater than 1 year.
- Availability of panoramic radiographs.
- Presence of the contralateral premolars 4.4 and 4.5 in patients with agenesis of 3.4 and 3.5.

**Patients with any of the following characteristics were excluded**:

- Distorted uncalibrated radiographs.
- Caries and/or fillings in 7.4 and 7.5.
- Bilateral agenesis of the lower premolars (3.4, 3.5, 4.4 and 4.5).

### Ethical considerations

A single blinded operator from the Department of Dental Clinical Specialties of UCM evaluated the radiographs of each patient. The measurements were made using digital X-ray with a computer. A maximum of 20 panoramic radiographs were evaluated per day to avoid operator fatigue. The operator was an experienced dentist previously trained in diagnosis by X-rays. Medical records were evaluated by Professor De Nova and Professor Mourelle. The study was approved by the Ethics Committee of the San Carlos Clinical Hospital of Madrid with the internal code 21/418-0_M_O, and the ethical criteria formulated by the Declaration of Helsinki of the World Medical Association on ethical principles for medical research on human beings were met.

Tooth development of the premolars and root resorption of the temporal molars.

The third quadrant was studied, focusing on the first left temporary molar (7.4), the second left temporary molar (7.5), the first left permanent premolar (3.4) and the second left permanent premolar (3.5). In patients with agenesis of 3.4 and 3.5, the contralateral premolars in the fourth quadrant, the first right premolar (4.4) and the second right premolar (4.5), were studied.

The *Demirjian* method was used to study the *dental development* (DD) of 3.4 and 3.5. This method is based on the progression of mineralization of each tooth, and the shape does not depend on length estimates [22]. Eight stages are recognized for molars and premolars (stage A to stage H), where A corresponds to the mineralization of the cusps in the form of one or several cones without fusion and H corresponds to a fully developed tooth.

*Root resorption* (RR) of the mesial and distal roots of 7.4 and 7.5 was studied by the *Haavikko* method [23] as modified by Vuorimies et al. in 2017 [20]. This method establishes four stages of root resorption according to root length [20]. Each tooth was assigned the following score: 1, without root resorption; 2, ¼ of the root is resorbed; 3, half of the root is resorbed; and 4, ¾ of the root is resorbed or exfoliated. The mesial and distal roots of the primary molars were analysed separately, and the scores ranged from 2 to 8 for one molar and from 4 to 16 for two molars.

### Statistical analysis

Statistical analysis was carried out with *IBM-SPSS Statistics*^®^ version 25 (reference: IBM Corp. Released 2017. IBM SPSS Statistics v 25.0 for Windows; Armonk. NY. USA).

The statistical techniques and tests applied consisted of the following:

- In the groups with *N* > 50, the Kolmogorov‒Smirnov test was applied, while in the groups with *N* < 50, the Shapiro‒Wilk test was used.
- The quantitative variables are described using the usual measures of (a) centrality (mean and median) and (b) variability (observed range, standard deviation and interquartile range).
- For the comparison of the means among groups of different subjects (independent of each other), the nonparametric alternative of the Mann‒Whitney test was used since the variables did not meet normality criteria.
- The effect size was calculated to express the magnitude of the differences between groups. The effect size was expressed in R2 (scale: 0–1) so that it could be compared between different types of data (units of measurement) and between different statistical tests.
- Spearman’s correlation coefficient was used to explore the correlation between nonnormally distributed quantitative variables.

*p* values < 0.05 were considered to indicate statistical significance.

## Results

This study included a group of 26 patients with osteogenesis imperfecta and a control group of 395 healthy patients without systemic diseases. The ages of these 421 children ranged between 6.01 and 11.99 years, with a mean of 9.21 years (95% CI 9.05–9.37). A total of 69.2% of the participants in the study group had OI type I, and 30.7% had OI type III or IV, as shown in Table 1. The sample was reasonably balanced according to sex, with 52.5% (221 patients) boys and 47.5% (200 patients) girls. A total of 198 patients were included in the < 9-year-old group, and 223 patients were included in the > 9-year-old group, as shown in Table 2.

**Table 1:** Age and sex distributions of the study group and control group

	OI type I	OI type III	OI type IV	TOTAL
→ Study Group				
Participants	18	2	6	26
Sex (M/F)	9/9	0/2	4/2	13/13
Age				8,05 (+-1,67)
→ Control group				
Participants				395
Sex (M/F)				208/187
Age				9,29 (+-1,62)

**Table 2:** Sex distribution of the study group and control group according to age subgroup

	Study Group		Control Group	
	Boys	Girls	Boys	Girls
**< 9 years old**	10	9	104	75
**> 9 years old**	3	4	104	112
**TOTAL**	13	13	208	187

Delayed exfoliation of primary molars and tooth development were observed in boys compared to girls in both the study group and the control group (*p* = 0.05). In addition, a linear correlation with age and eruptive processes was also observed; the older the subject was, the greater the dental development of the premolars and the greater the root resorption of the primary molars in both groups (table 3).

**Table 3:** Dental development and root resorption in boys, aged between 6.01 and 11.98 years, and the corresponding *p* values

Number of subjects	Median/Mean (S.D.)			Mann‒Whitney Test		Effect size: R2
	Study group 13	Control group 208		/Values/	*P* values	
***DD TOTAL***	8.00/8.69 (2.81)	10.00/10.82 (2.44)		2.98**	0.003	0.040
***DD 3.4***	4.00/4.62 (1.32)	5.00/5.64 (1.21)		2.91**	0.004	0.038
***DD 3.5***	4.00/4.08 (1.55)	5.00/5.18 (1.30)		2.86**	0.004	0.038
***RR TOTAL***	6.00/6.85 (3.08)	12.00/10.50 (4.73)		2.32 *	0.020	0.033
***RR 7.4***	4.00/3.77 (1.64)	6.00/5.67 (2.43)		2.67**	0.008	0.034
***RR 7.5***	2.00/3.08 (1.71)	4.00/4.82 (2.43)		2.37 *	0.018	0.029

SD standard deviation; *= statistical significance; **= high statistical significance; DD dental development; RR root resorption

### Dental development of premolars in boys

**Fig. 1:**
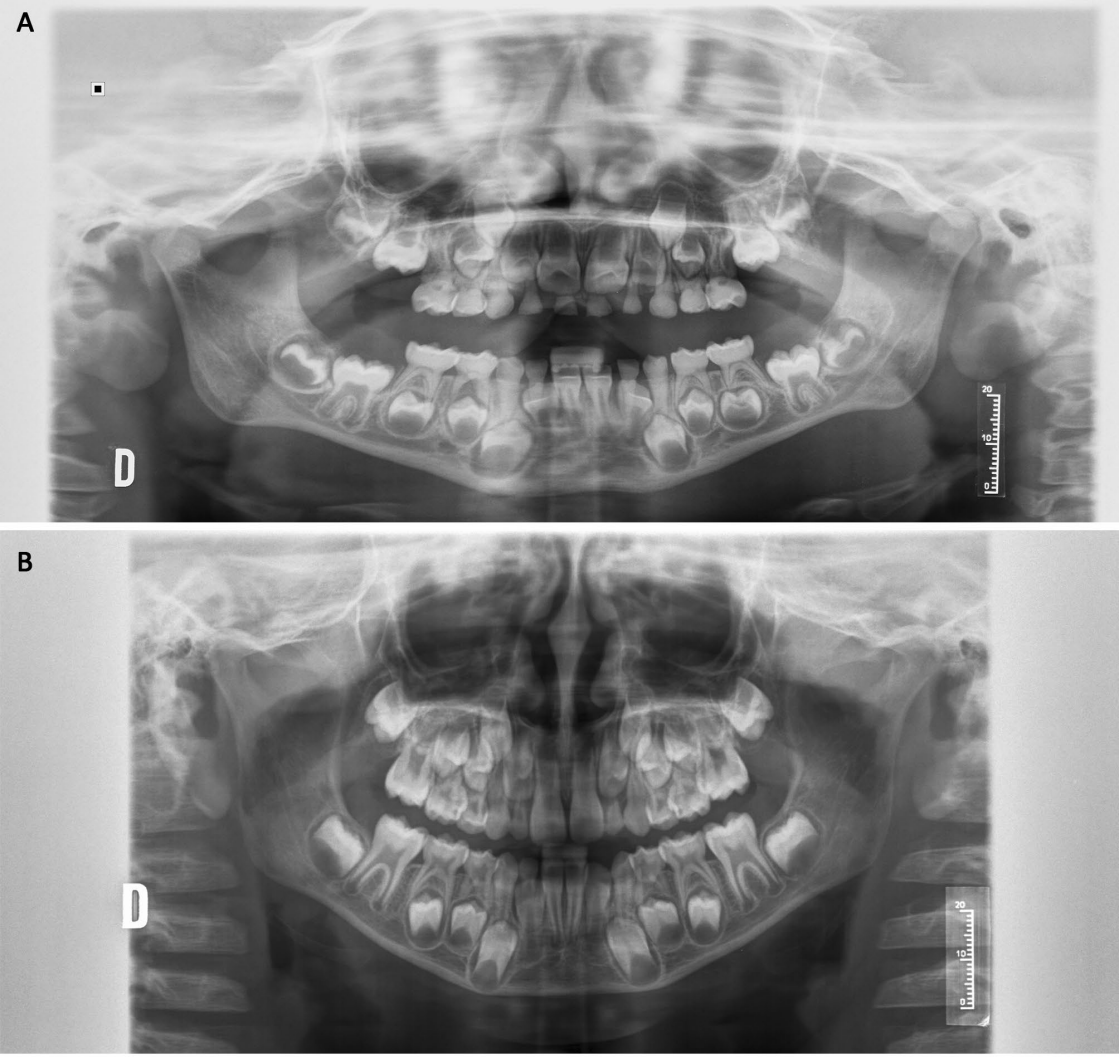
Two radiographs of 6.54-year-old boys. **A**. Patient in the study group with mixed dentition. Demirjian dental score for 3.4 and 3.5 = 7; RR score = 7. **B**. Control group patient with mixed dentition. Demirjian dental score for 3.4 and 3.5 = 10; RR score = 8

A significant delay in dental development was detected in boys with OI (*p* = 0.003). Figure 1 shows representative panoramic radiographs of two boys of the same age but exhibiting different eruptive processes. This difference remained in the group of boys under 9 years of age (*p* = 0.007). However, in boys over 9 years old, this difference was not maintained (*p* > 0.05), which could be influenced by the small sample size of this group. Therefore, we can conclude that the dental development of premolars in children with OI is decreased compared to that in the control group but is similar in boys over 9 years of age.

During the dental development of the premolars of boys with OI, crown formation was completed down to the cementoenamel junction (stage D), and the premolars of the control boys reached the beginning of root formation (stage E).

### Dental development of premolars in girls

**Fig. 2:**
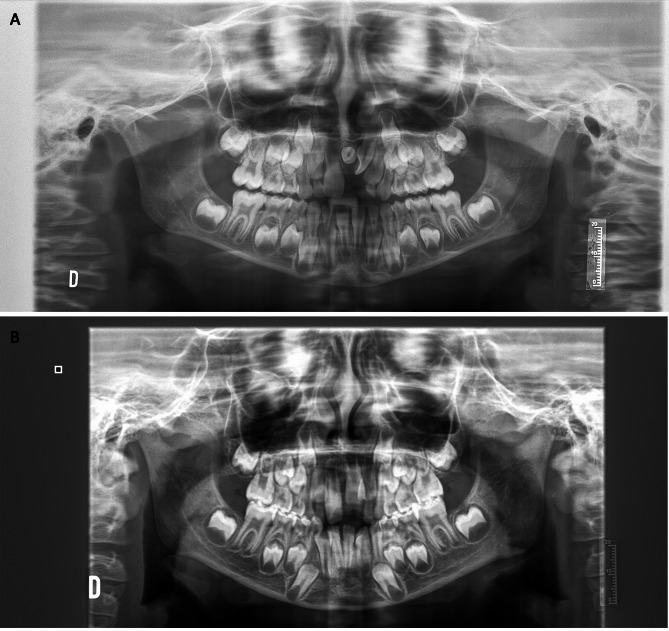
Two radiographs of 6.88-year-old girls. **a**. Patient in the study group diagnosed with mesiodens. Demirjian dental score for 3.4 and 3.5 = 9; RR score = 4. **b**. Control group patient. Demirjian dental score for 3.4 and 3.5 = 10; RR score = 4

Significantly delayed dental development was also described in the girls in the study group (*p* = 0.001). However, when we compared the girls by age group, in girls under 9 years of age, the values obtained were similar (p = > 0.05), and we cannot conclude that the dental development of the girls in the study group under 9 years of age was less than that of the control group. In the study group, delayed dental development of the premolars was significant in girls over 9 years of age (*p* = 0.027) (Fig. 2) (Table 4).

During the dental development of the premolars of the girls with OI had reached the beginning of root formation (stage E), and the root length of the premolars of the control girls was greater than the crown height (stage F).

**Table 4:** Dental development and root resorption in girls aged between 6.00 and 11.99 years and the corresponding *p* values

Number of subjects	Median/Mean (S.D.)			Mann‒Whitney Test		Effect size: R2
	Study group 13	Control group 187		/Values/	*P* values	
***DD TOTAL***	10.00/9.62 (1.39)	11.00/11.73 (2.29)		3.18**	0.001	0.051
***DD 3.4***	5.00/5.00 (0.82)	6.00/6.10 (1.18)		3.15**	0.002	0.052
***DD 3.5***	5.00/4.62 (0.65)	5.00/5.63 (1.18)		3.10**	0.002	0.045
***RR TOTAL***	6.00/7.15 (3.58)	14.00/12.14 (4.08)		4.02**	0.000	0.085
***RR 7.4***	4.00/4.54 (2.60)	8.00/6.52 (1.92)		2.87**	0.004	0.059
***RR 7.5***	2.00/2.62 (1.19)	6.00/5.63 (2.31)		4.51**	0.000	0.098

SD standard deviation; *= statistical significance; **= high statistical significance; DD dental development; RR root resorption

### Root resorption of primary molars in boys

The root resorption of 7.4 was 25% in boys in the study group and 50% in the control group. The root resorption of 7.5 was 25% in boys in the control group, but no resorption had occurred in the study group.

When analysing the exfoliation of the primary molars in both groups without stratification by age, delayed exfoliation was observed in the study group, and the difference was statistically significant (*p* = 0.020). However, when comparing the exfoliation of primary molars according to age, the results obtained were similar in both groups (*p* > 0.05) (Fig. 1). This result could have been influenced by the small sample size of the study group.

### Root resorption of primary molars in girls

In the girls in the control group, 7.4 was exfoliated, and the root resorption of 7.5 was 50%. The root resorption of 7.4 and 7.5 was beginning in girls with OI medicated with BPs.

Delayed exfoliation of primary molars was described in girls with OI, and the difference was significant (*p* = 0.000). However, when comparing exfoliation according to age group, the differences were not similar; in girls under 9 years of age, there was a clear statistically significant difference (*p* = 0.005). Analysis of the root resorption of primary molars 7.4 and 7.5 in girls over 9 years of age showed that the resorption of 7.5 was altered, as reflected in the total RR (*p* = 0.000). However, for the exfoliation of 7.4, the results were similar (*p* > 0.05) (Fig. 2).

## Discussion

Panoramic radiography is the main recommended screening method in dentistry; it is widely used in paediatric dentistry and is important for establishing a correct diagnosis [24]. A total of 421 panoramic radiographs were analysed to estimate the dental development of premolars and the root resorption of primary molars. The Demirjian method is one of the best known and most commonly used methods for estimating dental age, and it has been validated by numerous authors [25, 26]. One of the advantages of this method is that it allows the use of panoramic radiographs and does not require other invasive methods. Because the Demirjian method tends to overestimate dental age, dental age was not estimated in this study [26]. We used the Demirjian method to analyse and compare the dental development of the lower premolars between the groups.

Prove et al. reported a high incidence of uneven root resorption during the exfoliation process, with approximately 36% of the primary teeth demonstrating reduced resorption of one or more roots [27]. Peretz et al. reported that approximately 41% of root resorption in the primary first molar was symmetrical and approximately 56% of root resorption in the primary second molar was symmetrical [28]. In this study, root resorption was assessed based on the method described by Haavikko and modified by Vuorimeis et al. [20]. To perform the analysis in this study, the Haavikko method was modified to study the mesial and distal roots of the primary molars separately.

When analysing the entire sample without the subgroups, the results were very clear and indicated delayed dental development of the premolars and exfoliation of the primary molars in boys and girls with OI treated with BPs. These results support the hypothesis that children with OI treated with BPs have disruptions in the eruptive process and confirm the clinical findings of other authors [21, 29, 30]. 

Laganà et al. [31] reported that mesiodens was the most common type of supernumerary tooth detected on panoramic radiographs in nonorthodontic patients. In this study, this dental anomaly was found in one of the 26 patients in the study group. In contrast, in the control group, which consisted of 395 subjects, this dental anomaly was not observed. Taqi et al. described a greater prevalence of missing teeth in patients with OI than in the general population. This clinical finding was associated with the early administration of BPs, which seems to increase the risk of developing unerupted teeth [32]. Dental agenesis and morphological aberrations such as enamel defects are associated with the onset of medication administration before the age of 2 years [4, 21]. 

According to the rare and controversial published scientific studies, patients with OI medicated with BPs exhibited delayed dental eruption. Kamoun-Goldrat et al. [30]. reported a delay of 1.67 years in tooth eruption, Malmgren et al. [21]. reported a delay in tooth eruption, and a recent longitudinal study confirmed delayed eruption of the first-stage mixed dentition in children with OI medicated with BPs compared to a control group of healthy children; this delay was directly related to the cumulative dose of BPs [29]. However, Vuorimeis et al. [20]. reported no correlation between cumulative bisphosphonate dose or treatment length and any measured component of dental development [20]. 

As stated by Vuorimeis et al. [20]. OI was found to cause advanced dental development, and bisphosphonate treatment had a delaying effect, resulting in a rate of dental development that was indistinguishable from that of healthy children. The difference probably resided in the fact that in this study, the dental development of the seven left mandibular teeth was not analysed as other authors have reported [20]. In this study, the dental development of 3.4 and 3.5 was analysed individually and together. Observations in animal studies have demonstrated that bisphosphonate slows dental development [17–19]. Our results revealed that OI patients treated with BPs had significantly less dental development in premolars. Malmgren et al. [21]. reported delayed dental maturation in children treated with BP therapy before 2 years of age. In our study, all participants started treatment with BPs before 2 years of age.

Eruption of the premolars requires root resorption and exfoliation of the primary molars [33]. BPs act by blocking osteoclast function and thereby decrease bone resorption [12]. A direct relationship between bisphosphonates and the reduced ability of osteoclasts to resorb bone has been shown in animal studies [34]. The half-life of BPs is a decade, so the effects on bone persist throughout the entire eruptive process in children with OI medicated with BPs [3]. In the present study, we demonstrated a significant delay in root resorption of the primary molars in children with OI medicated with BPs. Vuorimeis et al. [20]. reported a significant delay in root resorption of the primary teeth in children with OI medicated with BPs compared to children with OI not treated with BPs. These results demonstrated a delaying effect of BPs on the resorption of primary teeth.

When we analysed the dental development of the premolars and root resorption of the primary molars according to subgroups of sex and age, these differences were not clearly apparent in all of the subgroups. Notably, in some subgroups, the sample size was small; therefore, the results must be interpreted with caution. There were two main reasons for the reduced sample size in the study group: OI is a rare disease with an approximate incidence between 6 and 20 cases per 100,000 live births, [1] and we excluded patients with caries, obturations, orthodontic treatments, and dental agenesis in the lower arch so all patients started with similar conditions and only the physiological process of dental development and root resorption of the primary teeth could be measured; 14 patients were excluded from the study group.

Malmgren et al. [21]. reported that dental maturity was significantly delayed in boys with OI who had been treated with BPs compared with healthy controls; these results were supported by our clinical findings of a significantly decreased dental development of the premolars and decreased root resorption of the primary molars in boys and girls with OI medicated with BPs (*p* = 0.05). Over the last century, puberty has been studied in both girls and boys. Epigenetic, nutritional, genetic, hormonal and environmental factors may also be involved in the eruptive process [35]. In this study, in girls over 9 years of age, no difference was found in the exfoliation of 7.4, perhaps because the resorption of the first primary molar begins at 7 years of age, [28] and by 9 years of age, exfoliation has already occurred in most patients.

To the best of our knowledge, there are no published studies analysing the individual dental development of premolars and root resorption of primary molars in children with OI who were strictly matched for sex and age. The study design is cross-sectional, and only associations can be established. We cannot discern whether the alteration in the eruptive process was due to BP treatment or osteogenesis imperfecta.

This study did not include a group of patients with OI without medication since OI patients receive cycles of BPs at very early ages in Spain per protocol. Among all the patients who were recruited for the study, only one patient did not use antiresorptive medication, and this patient was subsequently excluded for not being medicated for 1 year or more. Interestingly, in Scandinavian countries, there are groups of patients with OI without medication [20]. These studies are very clear and indicate that BPs should be administered only for severe OI and not for mild OI. In children with mild OI, only antiresorptive agents should be administered if a clear clinical benefit can be demonstrated in controlled clinical trials [36].

Our study showed that children with OI who received BP therapy had delayed root resorption and dental development. Clinicians should be aware of this delay because it may increase the number of impacted teeth in children who already suffer from dental disorders.

## Conclusions

Children with OI medicated with BPs exhibit delayed dental development of the premolars and delayed root resorption of the primary molars. According to sex, both variables were delayed in boys, but the difference did not remain when data were analysed by age subgroup. These clinical findings support the importance of clinically and radiographically monitoring the dental development and root resorption of primary teeth in children with OI treated with BPs to avoid alterations of the eruptive process.

### Electronic supplementary material

Below is the link to the electronic supplementary material.

Supplementary Material 1

Supplementary Material 2

Supplementary Material 3

## Data Availability

The data that support the findings of this study are available at the Complutense University of Madrid, but restrictions apply to the availability of these data, which were used under licence for the current study and are not publicly available. However, the data are available from the authors upon reasonable request and with the permission of the Complutense University of de Madrid. Clara Garcete (cgarcete@ucm.es) should be contacted to request the data from this study.

## Abbreviations

OI: Osteogenesis imperfecta
BPs: Bisphosphonates
RR: Root resorption
DD: Dental development
